# Astaxanthin Suppresses MPP^+^-Induced Oxidative Damage in PC12 Cells through a Sp1/NR1 Signaling Pathway

**DOI:** 10.3390/md11041019

**Published:** 2013-03-28

**Authors:** Qinyong Ye, Xiaodong Zhang, Bixia Huang, Yuangui Zhu, Xiaochun Chen

**Affiliations:** Department of Neurology, Fujian Institute of Geriatrics, The Affiliated Union Hospital of Fujian Medical University, 29 Xinquan Road, Fuzhou, Fujian 350001, China; E-Mails: Xdzhang8411@163.com (X.Z.); bixiahuang1987@163.com (B.H.); zhuyg@nsfc.gov.cn (Y.Z.); chenxiaochun998@gmail.com (X.C.)

**Keywords:** astaxanthin, Parkinson’s disease, MPP^+^, PC12 cells, Sp1, NR1

## Abstract

Objective: To investigate astaxanthin (ATX) neuroprotection, and its mechanism, on a 1-methyl-4-phenyl-pyridine ion (MPP^+^)-induced cell model of Parkinson’s disease. Methods: Mature, differentiated PC12 cells treated with MPP^+^ were used as an *in vitro* cell model. The MTT assay was used to investigate cell viability after ATX treatment, and western blot analysis was used to observe Sp1 (activated transcription factor 1) and NR1 (NMDA receptor subunit 1) protein expression, real-time PCR was used to monitor Sp1 and NR1 mRNA, and cell immunofluorescence was used to determine the location of Sp1 and NR1 protein and the nuclear translocation of Sp1. Results: PC12 cell viability was significantly reduced by MPP^+^ treatment. The expression of Sp1 and NR1 mRNA and protein were increased compared with the control (*p <* 0.01). Following co-treatment with ATX and MPP^+^, cell viability was significantly increased, and Sp1 and NR1 mRNA and protein were decreased, compared with the MPP^+^ groups (*p <* 0.01). In addition, mithracycin A protected PC12 cells from oxidative stress caused by MPP^+^ by specifically inhibiting the expression of Sp1. Moreover, cell immunofluorescence revealed that ATX could suppress Sp1 nuclear transfer. Conclusion: ATX inhibited oxidative stress induced by MPP^+^ in PC12 cells, via the SP1/NR1 signaling pathway.

## 1. Introduction

Parkinson’s disease (PD) is characterized by the progressive degeneration of dopaminergic neurons in the *substantia nigra pars compacta*. Although the cause of PD has not yet been completely understood, several risk factors have been proposed in association with the etiology of the disease, including age, genetics, oxidative stress, mitochondrial dysfunction, and environmental factors [1]. However, the study of various markers and indices in PD patients and animal models indicates that increased reactive oxygen species (ROS) production caused by increased oxidative damage plays an important role in inducing neurotoxicity and eventually kills cells in PD [2]. Overproduction of ROS in Parkinson’s disease can lead to oxidative damage in the brain, as shown by increased lipid peroxidation, DNA damage and imbalance of iron ion metabolism in the *substantia nigra*. Increased protein oxidation is also apparent in many areas of the brain in Parkinson’s disease, particularly in the *substantia nigra* [3].

Astaxanthin (ATX), a non-provitamin A carotenoid found in the red pigment of shrimp, crab, salmon, and asteroidean, as a potent antioxidant, has been thought to provide health benefits by decreasing the risk of oxidative stress-related diseases [4]. Astaxanthin pretreatment was able to significantly inhibit apoptosis, mitochondrial abnormalities and intracellular ROS generation in either DHA-OOH- or 6-OHDA-treated SH-SY5Y cells [4]. ATX was able antagonize ischemia-mediated loss of aconitase activity and reduce glutamate release, lipid peroxidation, translocation of cytochrome c, and TUNEL labeling in the ischemic cortex, and did not alter physiological parameters, such as body temperature, cerebral blood flow, blood pressure, and pH [5]. In addition, a study found that ATX was able to cross the brain-blood barrier, suggesting that it may provide a neuroactive dietary compound [6].

Sp1 is a member of the Sp family of transcription factors that bind at GC boxes to regulate gene expression. Sp is usually an activator of transcription, upregulating downstream gene expression, and controlling cell growth and apoptosis. It is linked to the occurrence and progression of many diseases, including autoimmune disorder and malignant tumor [7]. *N*-methyl-d-aspartate (NMDA) receptors mediate fast excitatory synaptic transmission in the central nervous system and play a major role in neuronal processes such as synaptic plasticity, learning and memory. Overactivation of NMDA receptors can lead to neuronal degeneration thought to underlie various neurodegenerative disorders [8]. The formation of functional NMDA receptors (NMDARs) requires an essential NR1 subunit, one or more modulatory NR2 subunits (NR2A–D) and in some cases additional NR3 subunits (NR3A–B) [9]. During oxidative stress, Sp1 is upregulated, having downstream effects on NMDA receptor subunit expression [10]. The involvement of ROS and the nature of neuronal cell death in rodents is mainly apoptotic. Under oxidative stress, the upregulation of the NMDA receptor subunit NR1 may be an initiative factor in neuronal cell death [11]. Cell death induced by the lipid peroxidation product 4-hydroxy-2,3-nonenal (4HN) is attenuated by an NMDA receptor antagonist [12]. During hypoxic stress, the DNA binding activity of Sp1 is increased, leading to NR1 upregulation in the hippocampus, leading to cell apoptosis. Antioxidants attenuated the expression of Sp1 and NR1, as well as increased cell viability [7]. Pheochromocytoma (PC12) cells, derived from a clonal rat pheochromocytoma cell line, have been widely used as cellular models of Parkinson’s disease, as these cells share features with midbrain dopaminergic neurons. Rat PC12 cells express copious NR1 subunits of the NMDA receptor, but express very low levels of NR2C, with a complete absence of other types of NR2 subunits [13]. Therefore, PC12 cells are an ideal model for studying the relationship between Sp1 and NR1. ATX could markedly suppress 6-OHDA-induced apoptosis in human neuroblastoma SH-SY5Y cells, presumably through attenuating intracellular ROS generation, p38MAPK activation, and mitochondrial dysfunctions including the release of cytochrome c, the cleavage of caspase 9, caspase 3, and poly(ADP-ribose) polymerase (PARP) [3]. 

However, the role of the Sp1/NR1 signaling pathway in 1-methyl-4-phenylpyridinium (MPP^+^)-induced oxidative stress in PC12 cells has not been reported. Furthermore, whether ATX antagonizes MPP^+^-induced oxidative stress through the Sp1/NR1 cell-signaling pathway, has not been reported either. In this study, we used PC12 cells as a model of dopaminergic neurons, and caused cell injury with the neurotoxin MPP^+^. We observed whether ATX was protective and investigated its mechanism, finding that it may provide a promising candidate for chemoprevention and chemotherapy strategies for PD.

## 2. Results and Discussion

### 2.1. Cell Viability after MPP^+^-Induced Toxicity in PC12 Cells

**Figure 1:**
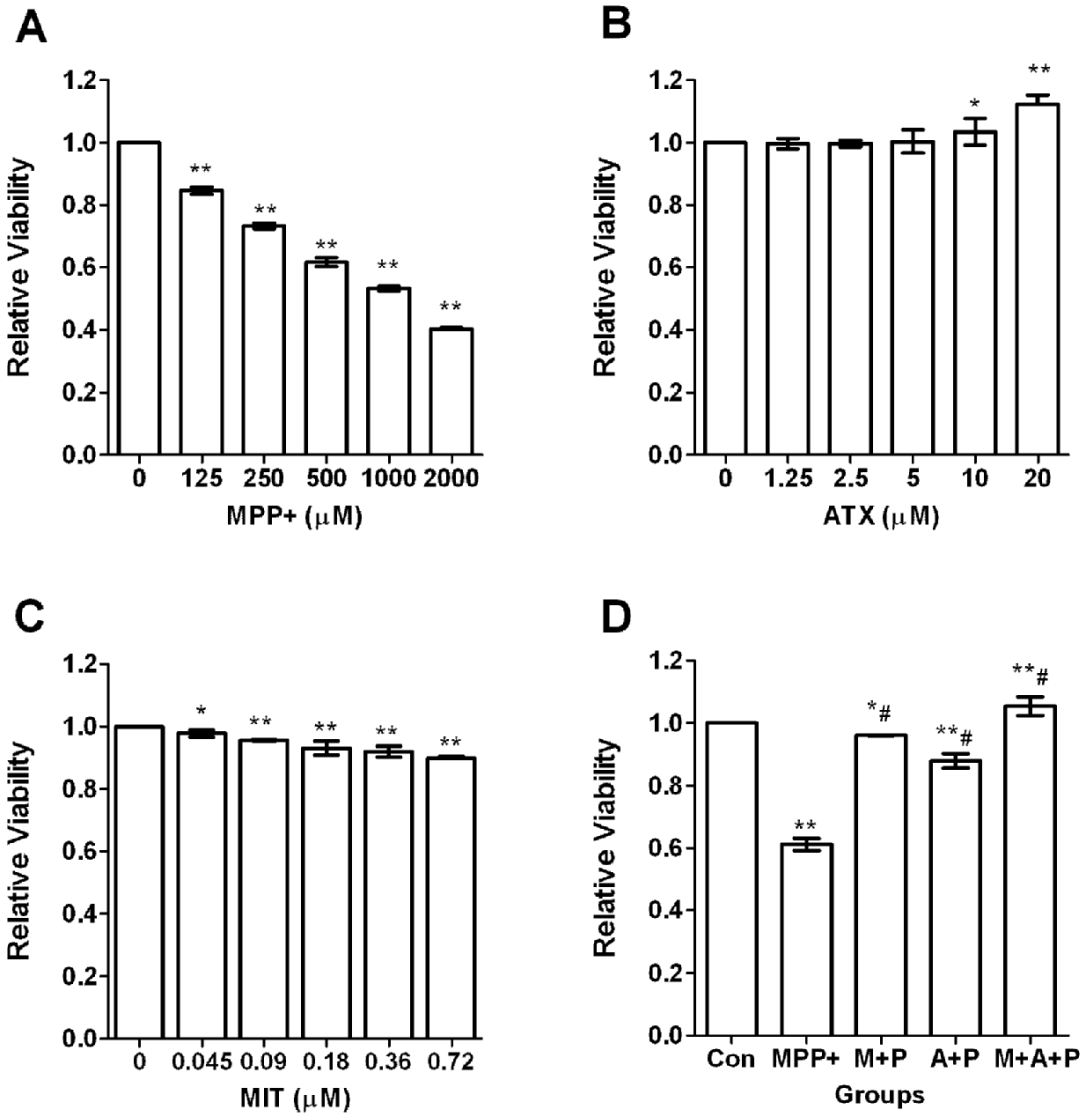
Astaxanthin (ATX), Mithramycin A (MIT) and 1-methyl-4-phenyl-pyridine ion (MPP^+^) effect on cell viability of PC12 cells. (**A**) The effect of MPP^+^ on cell viability in PC12 cells. The concentrations of MPP^+^ were 0, 125, 250, 500, 1000, and 2000 μmol/L (compared with the vehicle control group, ** *p <* 0.01); (**B**) The effect of ATX treatment on cell viability in PC12 cells. The concentrations of ATX were 0, 1.25, 2.5, 5, 10, and 20 μmol/L (compared with the vehicle control group, * *p <* 0.05, ** *p <* 0.01); (**C**) The effect of MIT treatment on cell viability in PC12 cells. The concentrations of MIT were 0, 0.045, 0.09, 0.18, 0.36, and 0.72 μmol/L (compared with the vehicle control group, * *p <* 0.05, ** *p <* 0.01); (**D**) The effect of various treatments on cell viability. The groups were Con (control group), P (MPP^+^ 500 μmol/L group), M + P (MIT 0.36 μmol/L and MPP^+^ 500 μmol/L group), A + P (ATX 10 μmol/L and MPP^+^ 500 μmol/L group), M + A + P (MIT 0.36 μmol/L and ATX 10 μmol/L and MPP^+^ 500 μmol/L group). Viabilities were compared with the vehicle control group (* *p <* 0.05, ** *p <* 0.01) or compared with the MPP^+^ group (^#^ *p <* 0.01).

To evaluate the viability of differentiated PC12 cells after exposure to oxidative injury, the PC12 cells were treated with different concentrations of MPP^+^ (125–2000 μmol/L) for 24 h. Increasing concentrations of MPP^+^ treatment significantly decreased cell survival (Figure 1A). As an optimal MPP^+^ concentration, 500 μmol/L MPP^+^ was selected for subsequent experiments because cell viability was decreased by 38.3% (*p <* 0.01) (Figure 1A). We then investigated the neuroprotective effects of ATX. ATX alone did not have any cytotoxic effect at concentrations ranging from 1.25 to 20 μmol/L. However, only at concentrations at or above 10 μmol/L, did ATX display significant protection, and cell viability was increased by 3.46% (*p <* 0.01). Accordingly, 10 μmol/L ATX was selected for subsequent experiments (Figure 1B). Mithramycin A (MIT), a specific SP1-DNA binding inhibitor, could downregulate Sp1 protein and its downstream gene expression. Different concentrations of MIT treatment slightly decreased cell survival, in a negative dose-dependent manner (Figure 1C). As an optimal MIT concentration, 0.36 μmol/L MIT was selected for subsequent experiments because cell viability was decreased by 8.09% (*p <* 0.01) (Figure 1C). PC12 cells were pretreated with ATX (10 μmol/L) and MIT (0.36 μmol/L) in the medium for 2 h, individually or together, then exposed to 500 μmol/L MPP^+^ for 24 h. MTT assays indicated that co-treatment with ATX significantly increased cell survival by 26.77% (*p <* 0.01), and MIT increased viability by 34.94% (*p <* 0.01), compared with MPP^+^ alone. However, the cell survival of both treated groups was still significantly lower (*p <* 0.05) than that of the control group (Figure 1D). These findings indicated that ATX had significant protection against MPP^+^-induced cell death; however, ATX only protected normal PC12 cells slightly. 

### 2.2. ATX/MIT Protect against MPP^+^-Induced Toxicity in PC12 Cells by Reducing ROS

**Figure 2:**
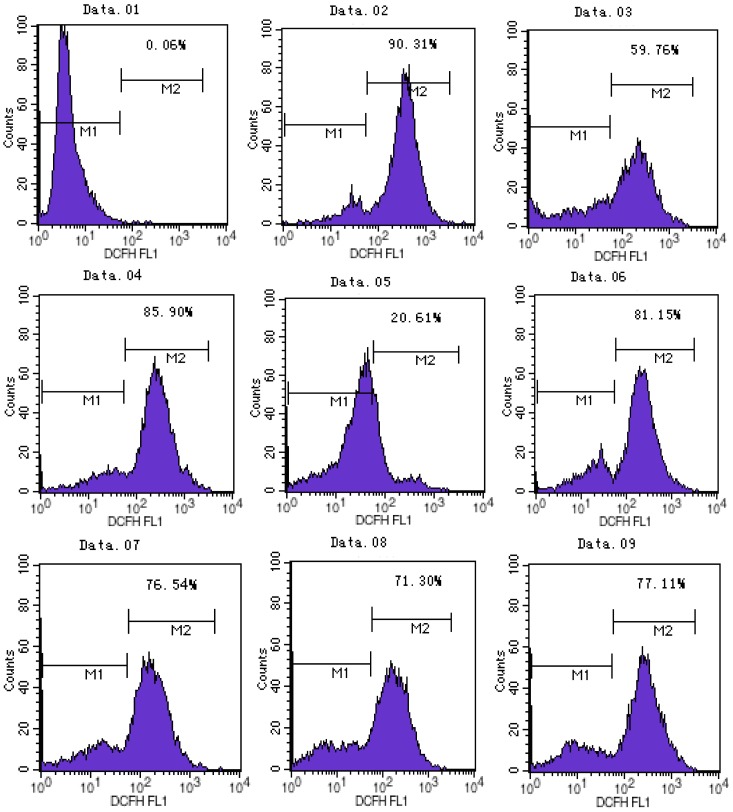
ATX and MIT protect PC12 cells against MPP^+^-induced toxicity by clearing ROS. The groups were: **01**, negative controlwithout 2′,7′-dichlorfluorescein-diacetate (DCFH-DA); **02**, positive control with Rosup; **03**, control; **04**, MPP^+^ 500 μmol/L; **05**, ATX 10 μmol/L; **06**, MPP^+^ 500 μmol/L and ATX 5 μmol/L; **07**, MPP^+^ 500 μmol/L and ATX 10 μmol/L; **08**, MPP^+^ 500 μmol/L and ATX 20 μmol/L; **09**, MPP^+^ 500 μmol/L and MIT 0.36 μmol/L. M1: negative control peak; M2:DCFH positive peak; Intracellular ROS generation was determined by DCFH-DA fluorescence.

The results of flow cytometry showed that ATX and MIT pretreatment could protect MPP^+^-treated PC12 cells from oxidative injury. ATX inhibited MPP^+^-induced oxidative damage in a dose-dependent manner. In the MPP^+^ group, ROS activity increased by 26.14%, compared with the control group. Compared with the MPP^+^ only group, the ATX-treated groups (5 μmol/L, 10 μmol/L, 20 μmol/L) showed decreased ROS activity (4.75%, 9.36%, 14.60%, respectively). In the MIT treated group ROS activity decreased by 8.79% (Figure 2).

### 2.3. The Expression Level of Protein Sp1 and NR1

**Figure 3:**
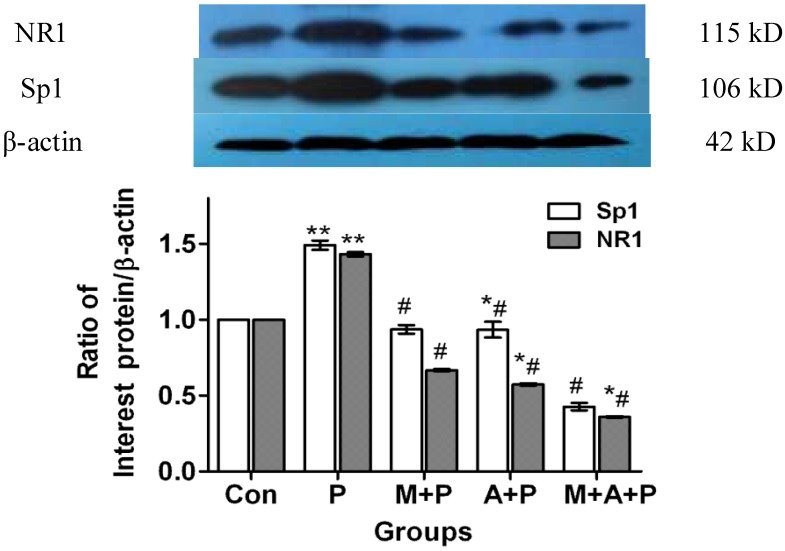
The effect of MPP^+^, MIT and ATX on expression of Sp1 and NR1 protein in PC12 cells, shown by western blot analysis. The groups: **Con** (control group), **P** (MPP^+^ 500 μmol/L group), **M** + **P** (MIT 0.36 μmol/L and MPP^+^ 500 μmol/L group), **A** + **P** (ATX 10μmol/L and MPP^+^ 500 μmol/L group), **M** + **A** + **P** (MIT 0.36 μmol/L and ATX 10 μmol/L and MPP^+^ 500 μmol/L group). MIT and ATX were also given as a 2-hour pretreatment before MPP^+^ treatment. Changes in Sp1 and NR1 protein expression were compared against β-actin as an internal reference. (Compared with the vehicle control group, * *p <* 0.05, ** *p <* 0.01; compared with MPP^+^ group, ^#^ *p <* 0.01; Image J analysis software).

Following western blot analysis, immunoreactive bands were present at relative molecular weights of 115 kD, 106 kD, 42 kD (Figure 3). Compared with the control group (using The Image J analysis software, National Institute of Mental Health, Bethesda, MD, USA), the expression of Sp1 and NR1 protein level in the MPP^+^ group increased by 54.07% (*p <* 0.01) and 54.66% (*p <* 0.01), respectively. Sp1 protein expression decreased by 1.39% (*p* > 0.05), and 13.23% (*p <* 0.01) in cells treated with MIT + MPP^+^ or MIT + ATX + MPP^+^ (respectively), and decreased by 1.30% (*p >* 0.05) in ATX + MPP^+^. NR1 protein expression in MIT + MPP^+^, ATX + MPP^+^, and MIT + ATX + MPP^+^ groups decreased by 1.71% (*p* > 0.05), 1.07% (*p* > 0.05) and 13.49% (*p <* 0.05) (Figure 3), respectively. Compared with the MPP^+^ only group, Sp1 protein expression in MIT + MPP^+^, ATX + MPP^+^, and MIT + ATX + MPP^+^ groups were decreased by 55.46% (*p <* 0.01), 41.03% (*p <* 0.01) and 67.30% (*p <* 0.01), respectively. NR1 protein expression in MIT + MPP^+^, ATX + MPP^+^, and MIT + ATX + MPP^+^ groups were decreased by 56.37% (*p <* 0.01), 55.73% (*p <* 0.01) and 68.14% (*p <* 0.01) (Figure 3), respectively.

### 2.4. The Expression Level of Sp1 and NR1 mRNA

**Figure 4:**
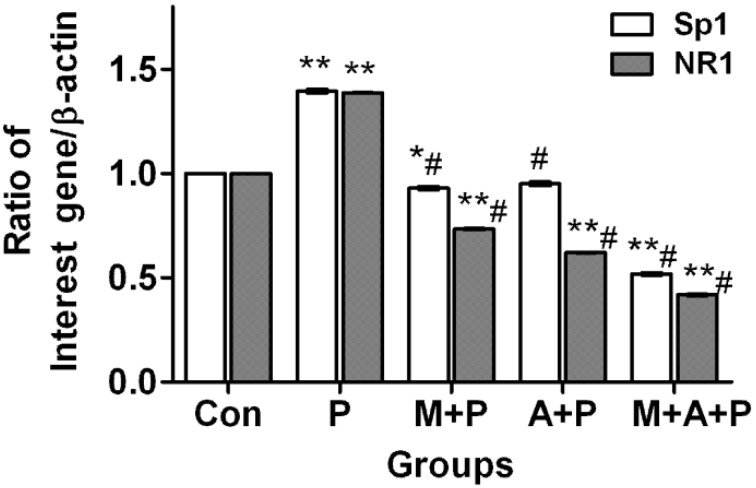
The effect of MPP^+^/MIT/ATX on expression of Sp1 and NR1 mRNA in PC12 cells with real time-PCR. Groups were: **Con** (control group), **P** (MPP^+^ 500 μmol/L group), **M** + **P** (MIT 0.36 μmol/L and MPP^+^ 500 μmol/L group), **A** + **P** (ATX 10 μmol/L and MPP^+^ 500 μmol/L group), **M** + **A** + **P** (MIT 0.36 μmol/L and ATX 10 μmol/L and MPP^+^ 500 μmol/L group). Two hour pretreatment with MIT and ATX affected Sp1 and NR1 mRNA levels (compared with the vehicle control group, * *p <* 0.05, ** *p <* 0.01; compared with MPP^+^ group, ^#^ *p <* 0.01).

The real-time PCR showed that Sp1 and NR1 mRNA levels in MPP^+^ treated cells increased by 39.57% (*p <* 0.01) and 38.85% (*p <* 0.01), respectively, compared with the control group. Sp1 mRNA expression in MIT + MPP^+^, ATX + MPP^+^, and MIT + ATX + MPP^+^ groups was decreased by 6.87% (*p <* 0.05), 4.75% (*p* > 0.05) and 48.46% (*p <* 0.01), respectively. NR1 mRNA expression in MIT + MPP^+^, ATX + MPP^+^, and MIT + ATX + MPP^+^ groups were decreased by 26.57% (*p <* 0.01), 37.87% (*p <* 0.01) and 58.09% (*p <* 0.01), respectively (Figure 4). Compared with the MPP^+^ group, Sp1 mRNA expression in MIT + MPP^+^, ATX + MPP^+^, and MIT + ATX + MPP^+^ groups was decreased by 46.45% (*p <* 0.01), 44.32% (*p <* 0.01) and 88.03% (*p <* 0.01), respectively. NR1mRNA expression in MIT + MPP^+^, ATX + MPP^+^, and MIT + ATX + MPP^+^ groups was decreased by 65.42% (*p <* 0.01), 76.72% (*p <* 0.01) and 96.95% (*p <* 0.01), respectively (Figure 4).

### 2.5. Localization of Sp1 and NR1 Protein, and Possible Nuclear Transfer of Sp1 in PC12 Cells

**Figure 5:**
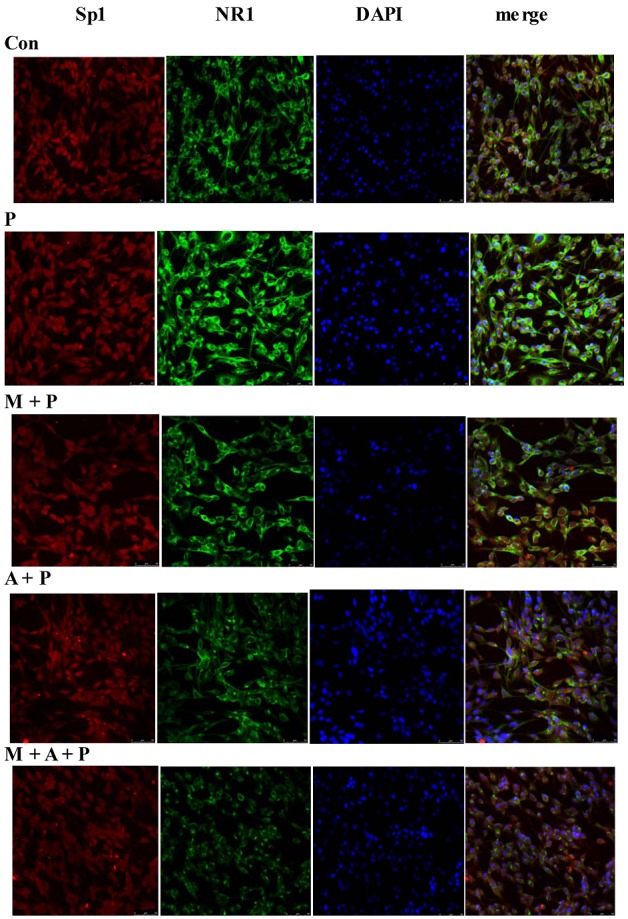
The localization of Sp1 and NR1 protein, as well as nuclear transfer of Sp1 in PC12 cells. The groups were: **Con** (control group, magnification 200×), **P** (MPP^+^ 500 μmol/L group, magnification 200×), **M** + **P** (MIT 0.36 μmol/L and MPP^+^ 500 μmol/L group, magnification 200×), **A** + **P** (ATX 10 μmol/L and MPP^+^ 500 μmol/L group, magnification 200×), **M** + **A** + **P** (MIT 0.36 μmol/L and ATX 10 μmol/L and MPP^+^ 500 μmol/L group, magnification 200×). Confocal microscopic images of the subcellular localization and expression of Sp1 and NR1. After the administration of MPP^+^ and other reagents to PC12 cells, Sp1 (Alexa Fluor 594, red fluorescence), NR1 (Alexa Fluor 488, green fluorescence), and nuclei counterstained with DAPI (blue) are shown.

Immunofluorescence microscopy showed that Sp1 (red) protein was found in both cytoplasm and nuclei, and NR1 (green) protein was mainly found in the cytoplasm of PC12 cells. In comparison with the control group, the expression of Sp1 and NR1 protein in the MPP^+^ group appeared to be significantly increased in the photomicrographs (Figure 5). The Sp1 and NR1 protein expression in MIT + MPP^+^, ATX + MPP^+^, and MIT + ATX + MPP^+^ groups were apparently decreased. Compared with the MPP^+^ group, Sp1 and NR1 protein expression in MIT + MPP^+^, ATX + MPP^+^, and MIT + ATX + MPP^+^ groups were also decreased (Figure 5). However, NR1 protein expression appeared to differ more in these groups. In addition, Sp1 protein changed not only in expression level, but also exhibited nuclear localization. Following MPP^+^ treatment for 24 h, Sp1 transferred from nuclei to cytoplasm. Two-hour pretreatment with MIT and ATX before the 24 h MPP^+^ treatment markedly reduced Sp1 nuclear transfer.

### 2.6. Discussion

The 1-methyl-4-phenyl-1,2,3,6-tetrahydropyridine (MPTP) model constitutes the best-characterized toxin paradigm for PD, clearly reflecting most of its clinical and pathological hallmarks [2,14]. It is believed that 1-methyl-4-phenylpyridinium (MPP^+^), which is the active metabolite of MPTP, inhibits mitochondrial complex I, causing abnormal energy metabolism and increased ROS production, finally resulting in a model that closely resembles PD [15,16]. PC12 cells, a clonal rat adrenal gland pheochromocytoma cell line, possess dopamine synthesis, metabolism, and transporting systems and retain the features of DA neurons and have therefore been used as an *in vitro* model for studies on PD [17]. In normal conditions, MPP^+^ generates several ROS, including O_2_^−^, H_2_O_2_, and ·OH [18], resulting in lipid peroxidation, DNA fragmentation, mitochondrial impairment, LDH leakage, GSH depletion [19], reduction of Na^+^K^+^-ATPase and catalase activities, increase of caspase-3 activity, and eventually cell death [20]. Thus, suppressants or inhibitors of MPP^+^-induced oxidative stress are considered to be potential agents for chemopreventive and chemotherapeutic strategies, though there are only a few studies regarding the protective effects of dietary factors on MPP^+^-induced apoptosis [21,22]. The present results showed that an abundantly available carotenoid, ATX, had protective effects on MPP^+^-induced apoptosis in PC12 cells and elucidated some of the underlying molecular mechanisms.

Increasing amounts of evidence suggests that MPP^+^-induced oxidative damage plays a primary role in the induction of neuronal apoptosis [23]. MTT results have reported that MPP^+^ apparently decreases PC 12 cells viability, ATX significantly inhibites MPP^+^-induced apoptosis, and MIT also suppresses PC12 cells death, which is in agreement with Lee D. H. and colleagues [24]. In addition, ATX at a concentration of 5 μmoL/L, exerts significant protective effects against MPP^+^-induced PC12 cell apoptosis, which presumably results from its antioxidant function [25]. Therefore, the initial blockage of ROS generation might be a very important factor for the protection of neurons. This study indicated that ATX also significantly reduced MPP^+^-induced intracellular ROS generation in a dose-dependent manner, thus, the downstream signaling pathway could be blocked [26]. Most importantly, Wang *et al*. recently found that glutamate-induced neurotoxicity in neuroblastoma cells and Aβ-induced apoptosis in PC12 cells were partly suppressed by ATX [27]. Furthermore, a study found that ATX decreased physiologically occurring oxidative stress and protected cultured cells against strong oxidative stress induced with a respiratory inhibitor [21], resulting in inhibition of nuclear factor kappa B-dependent gene expression. From these data, we speculate that, at least in part, ATX prevents MPP^+^-induced apoptosis through the mitochondrial-protection and modulating gene expression.

Free radicals can damage cellular lipid, protein and DNA, leading to cellular degeneration and finally death. In MPP^+^-induced oxidative stress, Sp1 mRNA and Sp1 protein increase markedly. Following Sp1 overexpression, Sp1 binding to two GC-boxes was enhanced [28], leading to upregulation of many downstream genes, including oxidative and anti-oxidative genes. The subsequent effect mainly depends on the activated signal pathway. NR1 mRNA and NR1 protein are also similarly upregulated [29]. When NR1 protein is increased, there is a resulting increase in the cellular response to NMDAR stimulation and diminished monolayer impedance. Oxidative stress upregulates NMDAR on the cerebrovascular endothelium and thus heightens susceptibility to glutamate-induced blood-brain barrier disruption, ultimately causing nerve cell degeneration [30]. Administration of a selective NMDA antagonist during exposure was found to ameliorate neuronal degeneration, providing evidence of excitotoxicity in oxidative stress [31]. As we can see from this *in vitro* study, pretreatment with ATX for 2 h can significantly inhibit MPP^+^-induced production of intracellular ROS and cytotoxicity in PC12 cells. Western blotting, real-time PCR and cell immunofluorescence have all confirmed that Sp1 and NR1 expression were upregulated by MPP^+^, Sp1 transferred from nuclei to cytoplasm and that pretreatment with ATX markedly suppressed this upregulation and Sp1 nuclear transfer. These results suggest that the neuroprotective effects of ATX are related to anti-oxidant activities following MPP^+^-induced oxidative damage [32]. Another study found that dietary astaxanthin could also enhance immune responses in young healthy females by decreasing a DNA damage biomarker and acute phase protein [33]. After treatment with MPP^+^, the Sp1 activity increased [34]. Mithramycin A and Sp1 are susceptible to oxidative stress induced by MPP^+^ [35]. Mithramycin A can specifically suppress Sp1 expression, thereby suppressing NR1 expression, and protecting PC12 cells from MPP^+^-induced oxidative damage and death. One study reported that the neuroprotective effect of mithramycin A was related to its ability to bind to GC-rich DNA and globally displaced Sp1 family transcription factors [36]. The authors also stated that in a MPP^+^-induced PC12 cell model, ATX and Mithramycin A exert a synergistic effect, and that these mechanisms need further study.

## 3. Experimental Section

### 3.1. Reagents

Cell culture reagents were purchased from Hyclone (Logan, UT, USA). Acrylamide and western blot reagents were purchased from Bio-Rad (Bio-Rad, Hercules, CA, USA). MPP^+^ (D048) and mithramycin A were purchased from Sigma-Aldrich (St. Louis, MO, USA). Astaxanthin (NO. 013-23051) was purchased from Wako Catalog (Wako, Tokyo, Japan). Sp1 (sc-420) and NR1 (sc-1467) antibodies were purchased from Santa Cruz Biotechnology Inc. (Santa Cruz, CA, USA). Actin antibody, mouse anti-goat IgG and goat anti-mouse IgG were purchased from Beijing Zhongshan Golden Bridge Biotechnology Company (Beijing Zhongshan Golden Bridge Biotechnology Co., Ltd., Beijing, China). Sp1 (NC_005106.2) and NR1 (NC_005102.2) primers were synthesized by Shanghai Biological Engineering Company (Shanghai Biological Engineering Company, Shanghai, China). Labeled Donkey Anti-Goat IgG Antibodies (A11055, Alexa Fluor 488, green) and Labeled Donkey Anti-Mouse IgG Antibodies (A21203, Alexa Fluor 594, red) were purchased from Invitrogen (Invitrogen, Carlsbad, CA, USA). 3-(4,5-Dimethylthiazole-2yl)-2,5-diphenyl tetrazolium bromide thiazolyl blue (MTT) were purchased from Wuhan Ling fly Technology Company (Wuhan Ling fly Technology Co., Ltd., Wuhan, China). The remaining reagents were purchased from Beyotime Company of Biotechnology (Beyotime Institute of Biotechnology, Shanghai, China).

### 3.2. Cell Culture

Highly differentiated PC12 cells, which were obtained from the Chinese Academy of Sciences Committee Type Culture Collection cell bank, were cultured in 100 mm tissue culture plates in high glucose DMEM supplemented with 10% (v/v) fetal bovine serum and 100 U/mL penicillin and 100 U/mL streptomycin. Cells were incubated at 37 °C in a humidified incubator (Forma Scientific, Marietta, OH, USA; Model No. 3130) containing 5% CO_2_. When the cells were 80%–90% confluent they were harvested and dispersed. The well-dispersed cells were then cultured for 24–36 h with ATX or an antagonist in the presence or absence of MPP^+^. The cultured medium was changed every 2–3 day. In some experiments, cells were pre-treated for 2 h with 10 μmol/L ATX and/or 0.36 μmol/L MIT, and stimulated with MPP^+^ (500 μmol/L) for 24 h. Control cells were cultured without MPP^+^.

### 3.3. MTT Assay to Evaluate Survival Cells

MTT is absorbed into cells, and then converted to formazan by mitochondrial succinate dehydrogenase. Accumulation of formazan reflects the activity of mitochondria directly and the cell viability indirectly. Cells were plated at a density of 1 × 10^4^ cells/well in 96-well plates, cultured, differentiated and treated according to the above described methods. PC12 cells were treated with various concentrations of ATX (1.25, 2.5, 5, 10, 20 μmol/L) or MPP^+^ (125, 250, 500, 1000, 2000 μmol/L) for 24 h *in vitro*. A total of 20 μL of MTT was added as a concentration of 0.5 mg/mL after media (200 μL) was added in each well. The 220 μL of solution was removed from each well, and then 150 μL dimethyl sulfoxide (DMSO) was added in each well. Optical density (OD) was evaluated at 570 nm on the ELISA plate reader after the precipitate in the well was dissolved on the microplate mixer for 10 min. All of results were zeroed against the OD measured in the same conditioned well without cell culture.

### 3.4. ROS Test

The 2′,7′-dichlorfluorescein-diacetate (DCFH-DA) assay was used to measure ROS production in differentiated PC12 cells treated with MPP^+^. DCFH-DA is a fluorescent dye that crosses the cell membrane and is enzymatically hydrolyzed by intracellular esterases to non-fluorescent DCFH. PC12 cells were seeded in six-well plates at a concentration of 10^5^ cells per well. After the cells were treated with MPP^+^ and/or ATX/MIT, the medium was removed, and the cells were washed three to four times with DMEM. The cells were incubated with DCFH-DA at a final concentration of 10 μmol/L in high glucose DMEM without FBS for 20 min at 37 °C and washed three times with DMEM. ROS levels were measured using a flow cytometer (FACScalibur, San Jose, CA, USA) with excitation and emission wavelengths set at 485 and 535 nm, respectively. For each analysis, 10,000 events were recorded. The value for each treatment group was converted to a percentage of the control value. 

### 3.5. Immunoblot Analysis

PC12 cells, plated at a density of 4 × 10^5^ cells per six-well dish, were treated with various concentrations of antagonist or ATX in media with or without MPP^+^ (500 μmol/L) for 24 h. The cells were washed with ice-cold phosphate buffered saline (PBS) several times, then the PBS was removed and cells harvested in RIPA Lysis Buffer (50 mM Tris pH 7.4, 150 mM NaCl, 1% Triton X-100, 1% sodium deoxycholate, 0.1% SDS, sodium orthovanadate, sodium fluoride, EDTA, 0.5 mM phenylmethanesulfonylfluoride (PMSF)). The lysates were incubated for 10 min on ice and centrifuged at 12,000× *g* for 10 min at 4 °C. The supernatant containing cell lysates was collected. Fifty micrograms of protein were loaded per lane, resolved by 10% SDS-PAGE (sodium dodecyl sulfate-polyacrylamide gel electrophoresis) for 90 min at 80–120 V. The separated proteins were transferred onto olyvinylidene fluoride (PVDF) membranes (Millipore, Carrigtwohill, Ireland) for 2 h at 300 mA with a Bradford reagent (Bio-Rad, Hercules, CA, USA). The membranes were blocked with 5% Skim milk in 1 × PBS (phosphate buffered saline) containing 0.05% Tween 20 (PBS-T) for four hours at room temperature. The membranes were then incubated with primary antibodies against Sp1 (1:100), NR1 (1:100) and β-actin (1:1000). Proteins were then detected with a horseradish peroxidase-coupled secondary antibody (Beyotime Institute of Biotechnology, Shanghai, China). Specific bands were visualized using the enhanced Chemiluminescence (ECL) detection kit. 

### 3.6. RNA Isolation and Real-Time PCR

Total RNA from PC12 cells was isolated according to manufacturer’s protocol using TRIzol reagent (Invitrogen, Carlsbad, CA, USA). Total RNA purity and integrity was confirmed by ND-1000 NanoDrop (NanoDrop Technologies, Wilmington, NC, USA) and 2100 Bioanalyzer (Agilent, Santa Clara, CA, USA). RNA (1 μg) was reverse-transcribed into cDNA in a total volume of 20 μL using the RevertAidTM First Strand cDNA Synthesis Kit (Fermentas, St. Leon-Rot, Germany). The cDNA (2 μL) was amplified with a sequence detection system (ABI Prism 7500) in a total volume of 20 μL containing 10 μL of the FastStart Universal SYBR Green Master Mix (ROX) (Roche, Penzberg, Germany) and each primer at 0.3 μM. Quantitative real-time PCR was performed using the ABI prism 7500 HT sequence detection system (Applied Biosystems, Forster City, CA, USA) based on the 59-nuclease assay [37] for the various genes indicated and the housekeeping gene GAPDH. Relative expression was calculated using the ΔΔCt method [38], and passed the validation experiment. PCR amplification was carried out on cDNA equivalent to 10 ng of starting mRNA using specific oligonucleotide primers for 

     Sp1 (forward, 5′-TGTGAATGCTGCTCATCAACTGTC-3′,

     Reverse, 5′-CAGGGCTGTTCTCTCCTTCTT-3′), and

     NR1 (forward, 5′-GCTGGGATTTTCCTCATTTTC-3′,

     Reverse 5′-GGGCTCTGCTCTACCACTCTT-3′), and

     β-actin (forward, 5′-GGAGATTACTGCCCTGGCTCCTA-3′,

     Reverse 5′-GACTCATCGTACTCCTGCTTGCTG-3′).

The conditions for PCR were initial denaturation for 3 min followed by 95 °C for 1 min, annealing temperature for 1 min 30 s, and 72 °C for 1 min 30 s for 40 cycles and a final incubation at 72 °C for 10 min. The PCR product thus obtained was loaded on a 2% agarose gel and visualized by ethidium bromide staining. The bands obtained were documented for densitometry. The experiments were performed in triplicate to ensure reproducibility of results.

### 3.7. Cell Immunofluorescence Technique

PC12 cells were permeabilized and fixed with 4% paraformaldehyde and 0.5% Triton X-100. Slides were blocked with 1% normal donkey serum (Merck, Darmstadt, Germany) in PBS for 30 min at room temperature. Cells were washed with 0.1% bovine serum albumin (BSA) (Beyotime Institute of Biotechnology, Shanghai, China)/PBS three times with gentle shaking, then incubated with the primary antibodies diluted (Sp1 1:50 and NR1 1:1000) in 0.1% BSA/PBS at 4 °C overnight. Labeled donkey anti-rabbit IgG or anti-mouse IgG (Invitrogen, Paisley, UK) (1:1000 dilution) was used as the secondary antibody and incubated in the dark for 2 h at room temperature. The cells were incubated with 0.5 μg/mL DAPI at room temperature for 30 min and mounted with anti-quenching medium. Specific antibody binding was detected by Alexa Fluor 488-(green label) and Alexa Fluor 594-(red label) conjugated extravidin (Sigma-Aldrich, St. Louis, MO, USA). Confocal microscopy was performed using the Leica SP5 confocal microscopy system (Leica Microsystems CMS GmbH, Mannheim, Germany). Optical sections were taken at 0.5 μm intervals and images were captured and stored digitally for analysis. Fluorescence intensity was quantified from at least three random fields (1024 × 1024 pixels; 310 × 310 μm) per slide, three slides per experimental condition, and repeated three times using separate cell cultures.

### 3.8. Data Analysis

All quantitative data were collected from at least three independent experiments. The final data are expressed as mean ± SEM. Differences between mean values were analyzed by one-way analysis of variance (ANOVA) and a *p* value < 0.05 was considered to be significant.

## 4. Conclusions

ATX as a potent antioxidant and has been thought to provide health benefits by decreasing the risk of oxidative stress-related diseases. This study showed that ATX at a concentration of 5 μmol/L exerted significant protective effects against MPP^+^-induced PC12 cell death, and ATX could specifically suppress Sp1 expression and Sp1 nuclear transfer, thereby suppressing NR1 expression. This study also found that ATX significantly reduced MPP^+^-induced intracellular ROS generation in a dose-dependent manner. In summary, we found that ATX exerts neuroprotective effects against MPP^+^-induced cell death and suppression of ROS production via the the inhibition of Sp1/NR1 signal pathway activation. ATX may also activate other pathways or protect PC12 cells by alternate mechanisms [39], which would require further study. These results suggest that ATX may provide a valuable therapeutic strategy for the treatment of progressive neurodegenerative diseases such as Parkinson’s disease.
